# Surgical applications of three-dimensional printing in the pelvis and acetabulum: from models and tools to implants

**DOI:** 10.1007/s00113-019-0626-8

**Published:** 2019-03-18

**Authors:** Christian Fang, Hong Cai, Evelyn Kuong, Elvis Chui, Yuk Chuen Siu, Tao Ji, Igor Drstvenšek

**Affiliations:** 1 Department of Orthopaedics and Traumatology, Queen Mary Hospital, The University of Hong Kong, Hong Kong, China; 2 Department of Orthopedics, Peking University Third Hospital, Beijing, China; 3 Department of Orthopaedics and Traumatology, Prince of Wales Hospital, The Chinese University of Hong Kong, Hong Kong, China; 4 Department of Orthopaedics and Traumatology, North District Hospital, Hong Kong, China; 5 Key Laboratory for Musculoskeletal Tumor of Beijing, Peking University People’s Hospital, Beijing, China; 6 Faculty of Mechanical Engineering, University of Maribor, Maribor, Slovenia

**Keywords:** 3D printing, Pelvis, Orthopaedics, Hip replacement, Pelvic tumour, 3-D-Druck, Hüfte, Orthopädie, Hüftgelenkersatz, Beckentumor

## Abstract

There are numerous orthopaedic applications of three-dimensional (3D) printing for the pelvis and acetabulum. The authors reviewed recently published articles and summarized their experience. 3D printed anatomical models are particularly useful in pelvic and acetabular fracture surgery for planning, implant templating and for anatomical assessment of pathologies such as CAM-type femoroacetabular impingement and rare deformities. Custom-made metal 3D printed patient-specific implants and instruments are increasingly being studied for pelvic oncologic resection and reconstruction of resected defects as well as for revision hip arthroplasties with favourable results. This article also discusses cost-effectiveness considerations when preparing pelvic 3D printed models from a hospital 3D printing centre.

## Zusammenfassung

Es gibt zahlreiche orthopädische Anwendungen des 3‑dimensionalen (3‑D) Drucks für Becken und Acetabulum. Im vorliegenden Beitrag werden in jüngerer Zeit publizierte Beiträge und eigene Erfahrungen zusammengefasst. Mittels 3‑D-Druck erstellte anatomische Modelle sind in der operativen Versorgung von Becken- und Acetabulumfrakturen besonders nützlich für die Planung, das Implantat-Templating und die anatomische Abklärung von Krankheitsbildern wie dem femoroacetabulären Cam-Impingement und seltenen Deformitäten. Mittels 3‑D-Druck maßgeschneiderte, patientenspezifische Metallimplantate und Instrumente werden vermehrt im Rahmen der onkologischen Beckenresektion und nachfolgenden Rekonstruktion von Defekten sowie in der Revisionsendoprothetik an der Hüfte getestet; die Ergebnisse sind positiv. Im vorliegenden Beitrag wird auch die Wirtschaftlichkeit diskutiert, wenn Beckenmodelle in einem innerklinischen Zentrum für 3‑D-Druck hergestellt werden.

## Introduction

Three-dimensional (3D) printing is increasingly applied in orthopaedics and traumatology [1, 2]. Surgery around the bony pelvis is challenging due to the complex anatomy, deep exposures and narrow safe corridors required to avoid critical neurovascular and visceral structures. The bony pelvis usually has high contrast compared to surrounding soft tissue on computed tomography (CT) scans and, therefore, is readily ‘segmentable’ into 3D models for virtual surgical planning using computer assisted design (CAD) and computer assisted manufacturing (CAM) techniques.

The authors conducted a review of the recent literature regarding 3D printing for bony pelvic and acetabular surgery. The search keywords ‘3d printing’, ‘rapid prototyping’ or ‘patient specific’ combined with ‘pelvis’ or ‘acetabulum’ were systematically searched in PubMed, and the retrieved articles relevant to the topic after screening for articles relevant to the question were discussed. Only articles published later than 2014 were included.

The authorsʼ (CF, HC, EC, TJ) hospitals have dedicated 3D printing services and a combined experience of over 400 cases for various surgical scenarios. In this article, they report the findings from the literature and combine the analysis with the report of their clinical experience using some case examples. 3D printing for pelvic and acetabular orthopaedic applications is discussed at three levels of complexity: firstly, anatomical models, secondly, non-implantable surgical tools and guides and thirdly, implantable prosthesis.

## Anatomical models

The simplest means of utilising 3D printing involves the fabrication of bone models. The first step called ‘segmentation’ describes a process whereby CT data, typically in digital imaging and communications in medicine (DICOM) format, is processed. The target anatomic structure, i.e., the bony pelvis in this case, is identified and converted into a digital 3D model usually in stereolithography (STL) format.

Virtual 3D models allow for the evaluation of 3D pathology, implant selection and basic virtual surgical planning by digital measurements and part manipulation. While software-based virtual 3D planning usually provides the surgeon with more information compared to using multiplanar CT image alone [3], model manipulation with 3D software is often non-intuitive to surgeons and intimidating.

3D printing rationalises virtual 3D planning by enabling surgeons to tactilely feel real-sized models

3D printing rationalises virtual 3D planning by enabling surgeons to tactilely feel real-sized models where scale, shape and anatomy are more effectively appreciated and collision between bone fragments is well represented [4, 5]. Models manufactured with materials fulfilling biocompatibility standards such as ISO-10993 can be sterilised following predefined protocols [6] and brought to the surgical field.

### Pelvic and acetabular fracture models

The most commonly reported technique utilises two 3D printed bone models, one of the ipsilateral fractured acetabulum and another of the mirrored intact acetabulum. Firstly, a full-scale replica of the ipsilateral pelvic fracture provides an accurate tactile impression of the volume, size and orientation of bone fragments. With an understanding of the fracture configuration, the best reduction technique, surgical approach and optimal screw trajectories are planned.

Secondly, the 3D printed mirror image of the opposite intact hemi-pelvis is invaluable in order for fixation plates to be accurately pre-contoured. Plate pre-contouring allows for better implant positioning and a reduction in surgeon fatigue. The optimal implant sizes, screw trajectories and lengths are determined preoperatively, and the need for implant repositioning is minimised.

Hung [7] conducted a retrospective comparative study of 30 patients with the above method and reported a 70-min reduction in surgical duration, a 270-ml reduction in blood loss, fewer complications and better radiological outcomes compared to conventional planning using CT images.

A meta-analysis by Zhang [8] of nine case-control studies consisting of 638 patients concluded that 3D printed bone models for surgical planning in pelvic and acetabular fractures resulted in a statistically significant reduction in surgical time, blood loss and the likelihood of inadequate fracture reduction compared to conventional imaging-based planning techniques. Similar benefits have been reported by various authors in smaller case series [7, 9–15] with minor variations in the techniques.

Chen [15] studied a minimalistic positive and negative 3D printed template of the bony surface. This is an intuitive approach to plate contouring with the implants ‘sandwiched’ between the two templates. The negative template also has predesigned drill holes for guiding the screw trajectories. In this study of 14 cadavers, 64 plates and 339 screws were placed with no hip joint penetrations. This method is advantageous in minimising time and material cost spent in 3D printing.

**Fig. 1:**
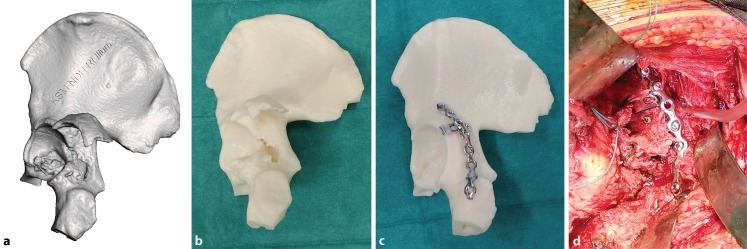
An example of a digital three-dimensional (3D) model of a posterior wall acetabular fracture in stereolithography format (**a**). The 1:1-sized 3D printed model allows the surgeon to accurately appreciate fracture morphology (**b**). A mirrored model is printed using the opposite intact hemi-pelvis for easy and accurate plate contouring (**c**). Surgical plan after fracture fixation (**d**). The implants are placed according to the surgical plan after fracture fixation reduction

Kim [11] described a technique in 14 patients where each major fracture fragment is printed separately and reduction is evaluated manually by using bone reduction tools as well as gluing them together. The optimal entry landmarks and trajectories for interfragmentary screws are located by simulated surgery under fluoroscopy. Alternatively, Zeng [13] reported a case series of 10 patients where fracture fragments are reduced digitally in the software. While this is more time consuming, it applies to situations with bilateral fractures or deformities when there is no intact contralateral pelvis to serve as a mirror reference (Fig. 1).

### Anatomical models for the deformed hip and pelvis

3D models are invaluable supplements to CT or magnetic resonance imaging (MRI) images in assessing various pathoanatomical situations. In CAM-type femoroacetabular impingement (FAI) osteoplasty, Wong [16] reported that 3D printed femoral and acetabular models allowed a dynamic appreciation of the site of impingement with the 3D printed femur and acetabulum models. Compared to conventional radiographic planning, nine out of 10 femurs and 10 out of 10 acetabula required a change in osteoplasty site. Childs [17] compared generic human hip models to 3D-printed models while counselling patients undergoing arthroscopic hip surgery for FAI and found a better understanding and retention of knowledge.

3D models are invaluable supplements to computed tomography or magnetic resonance imaging

**Fig. 2:**
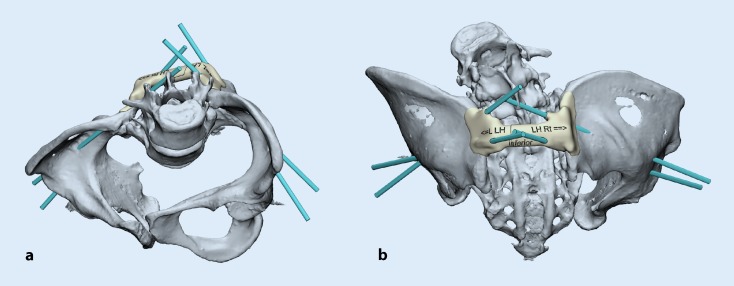
Design of a patient-specific drill guide for the placement of bilateral dual iliac screws for a patient with osteogenesis imperfecta undergoing posterior spinal fusion and instrumentation surgery in inlet (**a**) and posterior (**b**) orientations

**Fig. 3:**
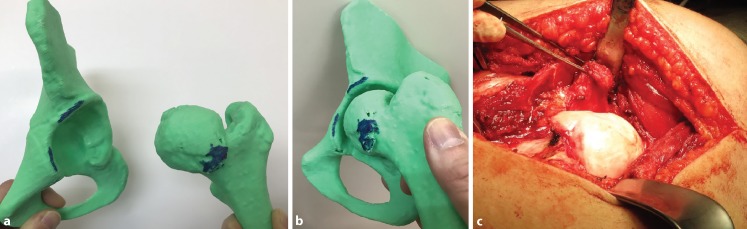
An example of showing the location and simulating mechanical femoroacetabular impingement in a 12-year-old patient. The offending CAM-type lesion is accurately located before osteochondroplasty, marked (**a**, **b**) and seen in surgery (**c**)

The authors [18] reported the successful use of a 3D printed bone model for accurate implant contouring before minimal invasive plate repair through an anterior approach at a fractured hip fusion site. Their centres find 3D printed models valuable for evaluating pelvic deformities in patients with skeletal dysplasia and neuromuscular conditions (Figs. 2 and 3).

## Three-dimensionally printed patient-specific tools and guides

The objective of using 3D printed patient-specific instrumentation (PSI) is to increase the precision of implant placement. The most commonly studied scenario is patient-specific guides for acetabular component socket preparation and installation in total hip arthroplasty (THA). The technique is recognised as an alternative to computer navigation. The use of PSI guides provides intraoperative time savings compared to computer navigation and is advantageous in reducing surgeon fatigue, anaesthetic duration and blood loss, but is arguably offset by increased resources spent in preoperative planning.

### Patient-specific instrumentation in primary total hip arthroplasty

Surgeons have mainly utilised PSI for, firstly, acetabular reaming and, secondly and more importantly, for guiding the orientation of the acetabular prosthesis. Buller [19] studied PSI THA for acetabulum component reaming and positioning in sawbone models. By using a cannulated reamer and a referencing guide pin for acetabular anteversion and inclination, PSI resulted in 9 degrees less variation in component orientation even in surgeons who operated on more than 1000 cases. In a cadaver study of THA, Sakai [20] showed that guide placement was within 1.0 and 1.7 degrees of optimal inclination and anteversion, respectively. However, a larger magnitude of error was later introduced during acetabular cup impaction with an error of 3.4 and 6.6 degrees of inclination and anteversion, respectively. Even with accurate placement of PSI tools, errors are still unavoidably introduced during manual impaction of the acetabular cups.

Small [21] conducted a prospective randomised study with the use of PSI THA reaming guides and a PSI-placed orientation pin. The technique resulted in significantly fewer anteversion errors while having no differences in inclination errors. Currently, there is no evidence to show that PSI results in improved clinical outcomes for primary hip replacements.

### Patient-specific instrumentation for pelvic tumour surgery and other scenarios

PSI cutting guides are valuable for tumour resection in the pelvis, allowing for accurate resection margins, graft sizing, opposition and, hence, improving the stability of the reconstructed pelvis [22] where complex, multi-faceted cuts are sometimes used in conjunction with custom-made endoprostheses. In a series of nine patients with 11 osteotomies reported by Gouin [23] for pelvic tumour resection, placement of PSI guides was usually straightforward in the pelvis with an accuracy of within 2.5 mm. As such, less soft tissue dissection is needed before osteotomy sites are identified correctly. Wong [24] compared PSI guides to computer navigation, showing a similarly clinically acceptable accuracy of 2.62 mm vs 3.6 mm at the resection planes, but required an average of 15 min less in a cadaver environment.

**Fig. 4:**
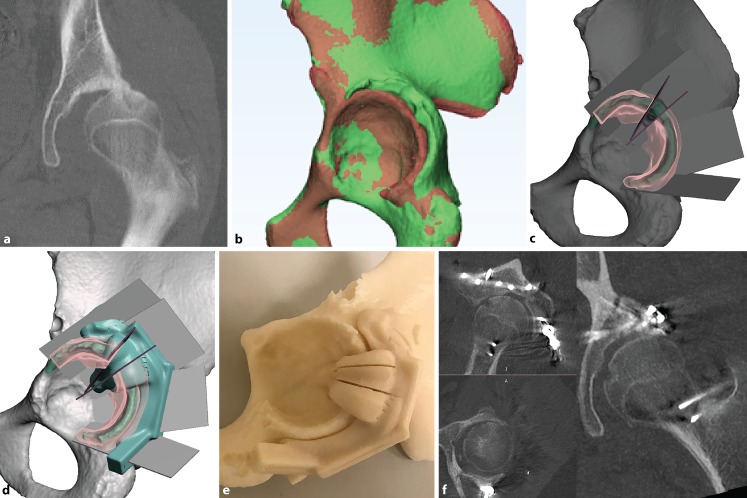
A 15-year-old patient with a malunited acetabulum posterior wall with hip subluxation (**a**). Digital model (*green*) compared to the mirrored opposite (*red*) (**b**). A closing-wedge volume-reducing osteotomy is planned (**c**) with 3D printed cutting jig (**d**, **e**). Postoperative computed tomography showing satisfactory restoration of hip congruency (**f**)

Zhou [25] reported a cadaveric evaluation of Bernese periacetabular osteotomies carried out using 3D printed surgical guides. The absolute precision of the correction of the lateral centre-edge angle was within 4 degrees compared to the preoperative plan. PSI is less commonly utilised for acetabular fracture repair; customised drill guides were studied by Merema [26] and successful in improving screw trajectories and minimising joint penetration in acetabular fracture surgery. The relative difficulty in reaching deeper bony landmarks makes PSI less favourable compared to percutaneous computer navigation and fluoroscopic guidance for intrapelvic screws. PSI is unable to reliably aid fracture reduction, which is still mostly manual and technical. In the authorsʼ limited experience, the application of PSI is valuable in recreating the fracture planes in complex intraarticular malunions of the acetabulum (Fig. 4).

## Metallic three-dimensional printing of customised implants

Customised implants have been used since the 1990s with success in the reconstruction of large-sized acetabular defects [27], tumour prostheses [28] and fracture plating [26, 29]. Close collaboration between the surgeon, technician and medical engineer is needed and considerable time and resources are dedicated to surgical planning. Typical production cycles are measured in weeks to months. Conventional computer numerical control (CNC) subtractive manufacturing techniques typically yield products with favourable longevity using high-quality alloy blocks. Constructs can be tested to be able to sustain physiological loads and optimised using finite element modelling (FEM) simulation before the design is finalised [30]. Many of these established CAD-CAM techniques are applied to the modern metal 3D printing workflow.

The use of custom-made titanium alloy implants manufactured by metal 3D printing technology, such as direct metal laser sintering (DMLS) or electron beam melting (EBM) technologies, is increasingly popular and arguably cheaper and faster. In keeping with the objectives of immediate and long-term stability, metal 3D printing can produce implants with complex shapes and porous internal structures controlled to the micrometre (µm) level for bony in-growth. Customisable textured surfaces and regional stiffness can minimise irritation to overlying soft tissues, stress concentration and stress shielding. 3D printed implants are typically implanted with PSI techniques and surgeons are provided with 3D printed models to aid resection and implantation. The technologies of 3D printing and computer navigation can be easily made complimentary, since 3D digital models in STL format are readily transferred between systems [30].

### Customised prosthesis for critical-sized pelvic and acetabular defects

A prime concern for metal 3D printed prosthesis is the unknown likelihood of fatigue failure compared to conventional CNC manufactured prosthesis, and this remains to be observed in larger case series and implant registries. Early reports of customised metal 3D printed implants for revision total hip arthroplasty are encouraging. Wyatt [31] reviewed a total of seven studies consisting of 243 custom-made hip replacement acetabular components for sizable Paprosky type III defects and pelvic discontinuities and showed a low likelihood of mechanical failures. In planning revision acetabular reconstruction surgery, the information on the 3D geometry of critically sized acetabular defects is better appreciated with PSI 3D planning techniques compared to using only radiographic grading following the popular Paprosky grading system.

Metallic 3D printed prostheses can reconstruct any part of the pelvis from the acetabulum, ilium and the sacrum. Liang [32] reported a series of 35 patients receiving customised modular 3D printed trabecular metal prosthesis where acetabular orientation and level can be adjusted intraoperatively. The early results were encouraging at 6–30 months with a low rate of complications. Angelini [33] reported the use of electron beam melting (EBM) fabricated titanium alloy prostheses for tumour excision in seven patients, again with encouraging results. In the above series, although usual complications and periprosthetic fractures were encountered, metallic failure of 3D printed prosthesis was not reported.

**Fig. 5:**
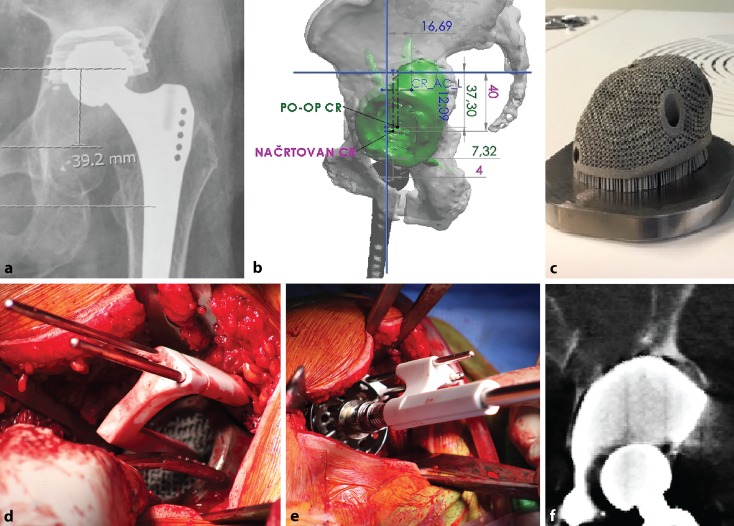
Patient with Paprosky IIIa defect and acetabular component loosening (**a**). Planning is performed using a digital three-dimensional model (**b**). Custom-made porous titanium alloy acetabular component printed using direct metal laser sintering (**c**). Intraoperative use of patient-specific instrumentation placed Schanz screws as positional references for implant (**d**) and reamer (**e**) placement. Postoperative computed tomography scan confirming satisfactory positioning (**f**)

**Fig. 6:**
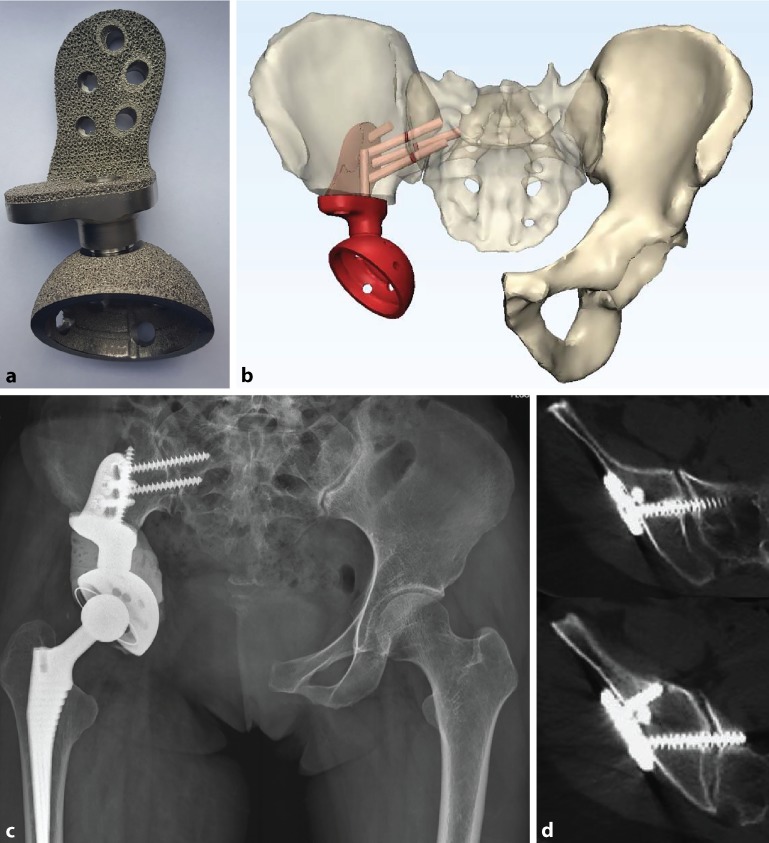
A 39-year-old patient with right acetabulum chondrosarcoma resection, reconstructed with an electron-beam melting three-dimensionally printed porous modular hemi-pelvic endoprosthesis (**a**) with preoperative virtual planning (**b**). Radiograph at 2 years (**c**), with computed tomography showing osteointegration (**d**)

The theoretical benefits of early stability and osseous integrations remain to be validated, and there is currently little evidence to tell whether routine use of 3D printed prostheses for the reconstruction of critically sized pelvic defects is cost-effective versus conventional means such as trabecular metal augments and structural allografts ([31]; Figs. 5 and 6).

## Hospital production of three-dimensional models

With printing done at the hospital, the team becomes aware of material and production time considerations and is likely to adopt an optimised workflow. Such a ‘Do-it-yourself’ approach without the support of engineers, sometimes using freeware and desktop 3D printers, has been reported to be successful [12, 34], with time and resource savings.

The five essential steps of 3D printing include segmentation, mesh optimisation, mesh verification, splicing and printing. Each of these steps can be performed by using either a combination of freeware or task-specific commercial software such as Mimics (Materialise, Leuven, Belgium). A commonly preferred freeware workflow utilises the 3D Slicer (https://www.slicer.org/) [35] for segmentation and meshes validation, Meshixer (Autodesk, San Rafael, USA) for model optimisation, mirroring and patient-specific tool creation, and Meshlab (http://www.meshlab.net) [36] for cavity filling.

Material extrusion, also called fused deposition modelling (FDM), is generally cheaper and requires fewer resources to operate than STL and laser sintering printers. Brouwers [37] compared the fabrication of pelvic bone models using low-cost desktop material extrusion 3D printers. The size inconsistencies were within 0.3%–0.8%—of good quality for surgical planning purpose.

In the authorsʼ experience, one challenge of 3D printing for pelvic and acetabular fracture lies in the relatively large size of pelvic bone models. The volume of substrate material and processing time is large relative to other types of bone models, hence increasing costs and the likelihood of delays in surgery. In Brouwers’ study [37], the material needed for printing a whole pelvis is 392–720 g and lasts 56–106 h. Smaller 3D printers are slower and do not always have the build volume to manufacture the full pelvis model in one go. Industrial grade 3D printers are generally more expensive to operate but more reliable, and time wastage is less likely when print jobs are aborted in mid-process. In the authorsʼ experience of using an industrial grade printer, a hollowed full pelvis model is produced at ‘low-quality settings’ in 22 h.

**Fig. 7:**
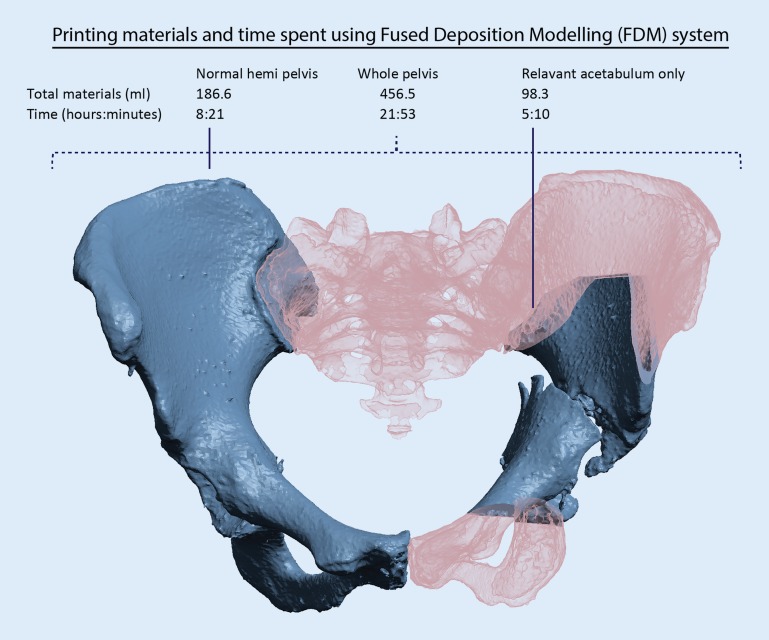
Using a fused deposition modelling (FDM) system at a 0.01-in. layer thickness (Fortus 450mc, Stratasys, Eden Prairie, MN, USA). Printing only the relevant fractured acetabulum results in 78% savings in materials and 76% savings in printing time compared to the whole pelvis

Cropping is recommended so that only relevant areas are printed. Compared to printing the whole pelvis, 50%–75% of materials and time are saved by cropping to only the relevant acetabulum. In the authorsʼ hospital-based 3D printing laboratory, the total processing time required from obtaining CT DICOM data to a fully processed and sterilised pelvic bone model typically requires no more than 24–48 h. Younger surgeons are encouraged to be personally in charge of the segmentation and modelling process while the 3D printer is operated by one technician. The authors believe this approach is the most workforce- and time-efficient and the software-related learning curve is quickly overcome (Fig. 7).

## Conclusion

For the pelvis and acetabulum, simple bone models are invaluable in planning fracture and femoroacetabular impingement surgery. PSI is studied with limited additional benefits for routine hip replacements, but useful in complex pelvic tumour resection and reconstruction. 3D printed metallic implants are increasingly used for the reconstruction of critically sized acetabular defects in complex revision hip replacement surgery and tumour endoprosthetic reconstruction with a favourable early safety profile.

The authors look forward to seeing a larger series of clinical studies, but guidelines are still lacking. In the coming decade 3D printing technology will become increasingly accessible. Surgeons must recognise and respect the different degrees of regulatory requirements imposed by different countries and implement necessary validation and quality assurance steps when using customised tools and implants.
